# Nigrosome 1 visibility at susceptibility weighted 7T MRI—A dependable diagnostic marker for Parkinson's disease or merely an inconsistent, age-dependent imaging finding?

**DOI:** 10.1371/journal.pone.0185489

**Published:** 2017-10-10

**Authors:** Carolin Gramsch, Iris Reuter, Oliver Kraff, Harald H. Quick, Christian Tanislav, Florian Roessler, Cornelius Deuschl, Michael Forsting, Marc Schlamann

**Affiliations:** 1 Department of Neuroradiology, University Hospital Giessen, Giessen, Germany; 2 Department of Diagnostic and Interventional Radiology and Neuroradiology, University Hospital Essen, Essen, Germany; 3 Department of Neurology, University Hospital Giessen, Giessen, Germany; 4 Erwin L. Hahn Institute for Magnetic Resonance Imaging, University Duisburg-Essen, Essen, Germany; 5 High Field and Hybrid MR Imaging, University Hospital Essen, Essen, Germany; Florey Institute of Neuroscience and Mental Health, The University of Melbourne, AUSTRALIA

## Abstract

**Background:**

Visualisation of nigrosome 1, a substructure of the healthy substantia nigra, was restricted in susceptibility weighted MR imaging in almost all patients with Parkinson's disease studied so far. The purpose of this study was to determine the degree of visibility of this substructure in subjects without Parkinson’s disease and to examine the potential link between increasing brain iron accumulation with age and its detectability.

**Methods:**

In 46 subjects (21 women, 25 men; 19 to 75 y; mean age: 44.5; SD: 15.6) examined with susceptibility weighted MR imaging at 7T visibility of nigrosome 1 was rated and classified. We assessed differences related to age and to signal intensities in the substantia nigra, red nucleus and putamen as correlates of the individual iron concentration.

**Results:**

In 93% nigrosome 1was at least unilaterally clearly present. In 24% at least one-sided limited visibility was observed. Using predefined classification criteria the specificity of the visibility across all age groups reached approximately 94%. We found no correlation with increasing iron concentrations with age.

**Conclusion:**

Aging with a related increase in iron concentration probably does not affect the visibility of nigrosome 1 at 7T SWI MRI. Our results support the role of this feature as a future differential diagnostic tool but further large-scale prospective studies are needed to better define the extent of a “limited visibility” to which an individual can be considered healthy.

## Introduction

Parkinson`s disease (PD) is the second most common neurodegenerative disease, only exceeded by Alzheimer`s disease, but parkinsonian features also occur as part of other diseases. Particularly the differentiation between PD and the various atypical parkinsonian disorders (APD) carries a high rate of misdiagnosis particularly in the early disease stages [1–4]. However, distinction between these entities is crucial because they differ in treatment response and prognosis [2, 3, 5, 6]. The main reason for the uncertainty in the diagnostic process is the lack of an established specific and dependable in vivo biomarker of nigral degeneration in PD. Recent imaging studies attempted to close this gap by assessing the substantia nigra (SN) on SWI- or T2*-weighted sequences for a marker which could mirror the loss of dopaminergic neurons [7––19]. For the first time studies examining iron related PD induced nigral changes did not focus on the whole SN or, at the most, on the subdivision into pars reticulate (SNr) and pars compacta (SNc) [7, 8] but on substructures of the SNc called nigrosomes. The largest of the five described nigrosomes, nigrosome 1, is localized in the caudal and mediolateral SN [7, 9, 10]. It contains the largest share of neurons which are most commonly affected in PD [7, 20]. In T2* weighted or in susceptibility weighted images (SWI) nigrosome 1 could be related to a cluster of high signal intensity [9, 12, 21–23] shaped like a “comma” or “wedge” [7] and surrounded by the low signal intensity of the SNc anterior and laterally and the medial lemniscus medially—a constellation which was associated with the tail of a swallow [7] and described as swallow-tail-sign (STS). Histological examinations detected a low iron and high neuromelanin content in this area [9]. Pathological studies of the dopaminergic subregions in PD revealed the first signs of cell loss in nigrosome 1 [11, 20]. Recent MR imaging studies reported that the STS was absent in PD, while healthy controls consistently displayed a hyperintense nigrosome 1 in iron-sensitive MR-imaging techniques [7, 9, 12–19]. Absence of the hyperintensity has been explained by the loss of dopaminergic neurons and increase of iron deposition in nigrosome 1 which is originally a structure with low iron content [9, 24, 25]. Studies so far have largely focused on visibility or absence of the nigrosome 1 itself or the swallow-tail-sign in patients with PD or additionally in patients with MSA and PSP. Assessment of the appearance in healthy subjects has been predominantly limited to the examination of small healthy control groups [7, 9, 12–19].

So far no study has focused on the visibility of nigrosome 1 in subjects without PD or other forms of degenerative neurological disease with regard to age-related changes in visibility.

In order to prove the reliability of this potential biomarker we retrospectively assessed the visibility of nigrosome 1 in all 7T SWI images of former UHF MR imaging studies with healthy subjects or those with diseases other than neurodegenerative disorders. We hypothesized that increasing brain iron accumulation in the aging brain might lead to a decreasing visibility also in subjects without PD. Thus, we measured the signal intensities of the substantia nigra (SN), nucleus ruber and putamen as assumed correlates of the individual iron content and compared them with the respective visibility of nigrosome 1.

## Materials and methods

### Subjects

All 7T UHF MR imaging examinations were carried out in the framework of studies approved by the ethics committee of our university (IRB: Ethik-Kommission der Medizinischen Fakultät Duisburg-Essen; Approval numbers: 06–3117 and 11-4898-BO). Written informed consent had been obtained from all subjects also concerning exploitation of their MR images within other studies. 46 study participants (21 women, 25 men) were included in the study. The age interval ranged from 19 to 75 years old (mean age = 44.5; SD = 15.6). All studies had been conducted from 2008 to 2012. Study specific exclusion criteria were any form of neurodegenerative parkinsonism like PD, MSA or PSP and ferritinopathies. All image sets originated from studies examinating either healthy persons or subjects with cavernomas, infarctions or tumors. Examinations were only excluded when these pathologies were located within the midbrain or at the level of the red nucleus or putamen.

### MRI examinations

All 46 subjects were examined on a 7T UHF whole-body research magnetic resonance system (MAGNETOM 7T, Siemens Healthcare, Erlangen, Germany) in combination with a 1-channel transmit/32-channel receive radiofrequency (RF) head coil (Nova Medical, New York, USA). The image acquisition was performed using a SWI 3D sequence with a repetition time (TR) of 29 ms, echo time (TE) of 15 ms, flip angle of 15°, slice thickness of 1 mm. The field-of-view was 168 x 224 mm, the imaging matrix was 896/672, resulting in a spatial resolution of 0.25 x 0.25 x 1.0 mm^3^. The acquisition time of the SWI sequence amounted to 14:3 min:sec to cover the entire brain. Standard axial slices were acquired parallel to a line linking the inferior borders of the genu and splenium of the corpus callosum.

### Image analysis

SWI images were assessed for the visibility of nigrosome 1 and the degree of signal intensity loss in the SN, posterolateral putamen and red nucleus. Furthermore all scans were scored for image quality. Image analysis was performed by two neuroradiologists, both of them with more than 5 years of experience in 7T UHF MR imaging. The examinations were evaluated on a PACS workstation (Centricity Radiology R 1000, GE Healthcare, IL, USA). Visibility of nigrosome 1 was scored independently by both observers to assess interrater reliability. Degree of signal intensity loss in the three nuclei and scan quality was measured and scored in consensus. Visibility of nigrosome 1 was rated for each hemi-mesencephalon separately on a scale from 0 to 2. A rating of 0 meant “not visible nigrosome 1”, 1 meant “not clearly delimitable” and 2 meant “clearly present”. In order to be able to calculate the inter-rater reliability between the two reviewers with Cohens kappa (κ) statistics we assessed every individual rating. Furthermore, in cases with different results we sought a consensus between both of them. According to A.I. Blazejewska et al. [9] to classify a hemi-mesencephalon as showing nigrosome 1 “clearly”, the hyperintense nigrosome 1 had to be visible as an ovoid substructure within the dorsal midbrain surrounded for more than half of its boundary by hypointense structures of the SN, not to correspond to a perivascular space and it had to be visible on at least two adjacent MR images. Afterwards, ratings of each side for each subject were classified into three groups according to Schwarz et al. [7]:

Group I (normal): Nigrosome 1 was clearly detectable bilaterally or unilaterally and contralateral possibly detectable
Group II (non diagnostic): Nigrosome 1 was bilaterally possibly detectable
Group III (abnormal): Nigrosome 1 was not detectable unilaterally or bilaterally.

**Fig 1:**
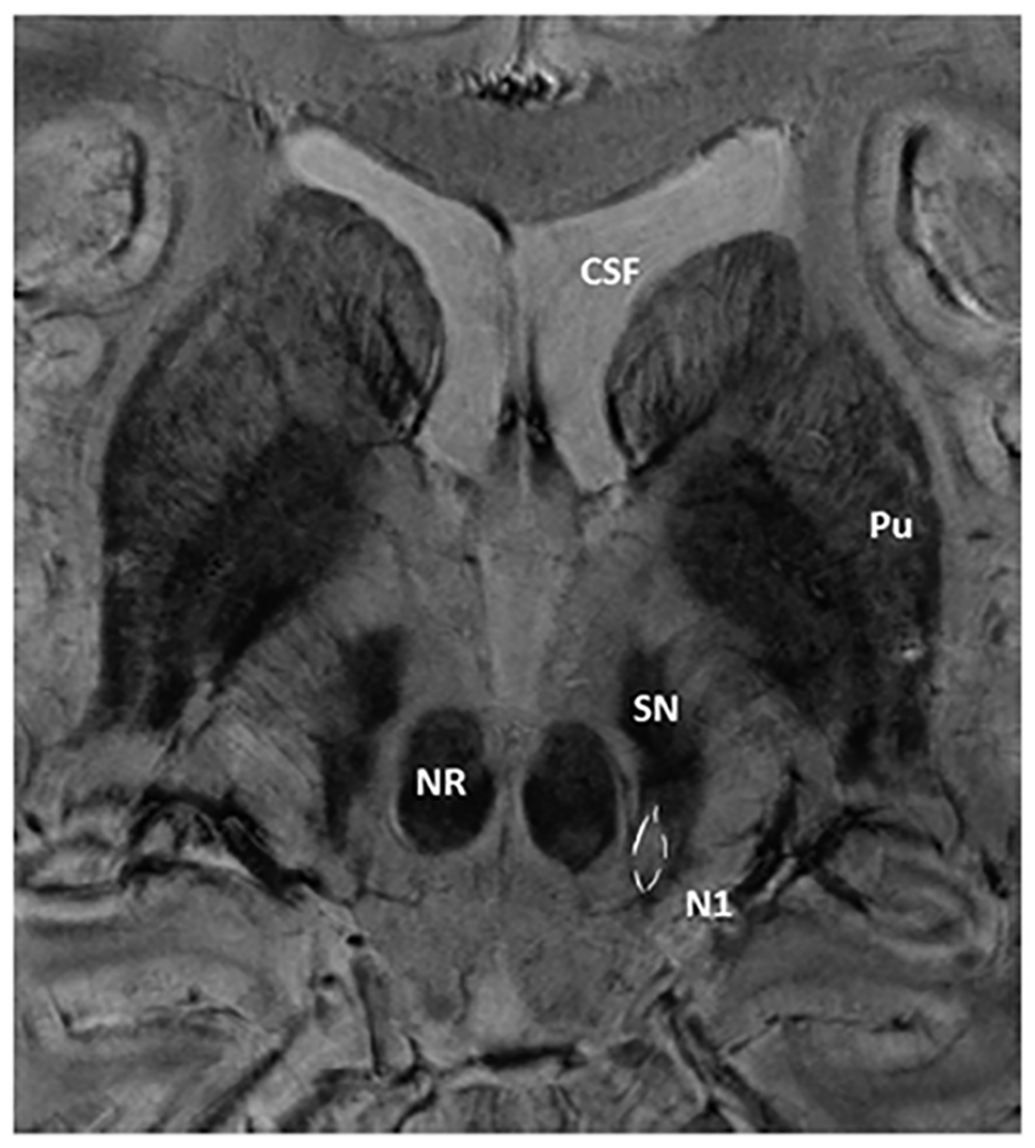
SWI 7T MRI of the midbrain and the surrounding structures. CSF: cerebrospinal fluid, Pu: putamen, SN: substantia nigra, NR: Nucleus ruber, N1: Nigrosome 1.

**Fig 2:**
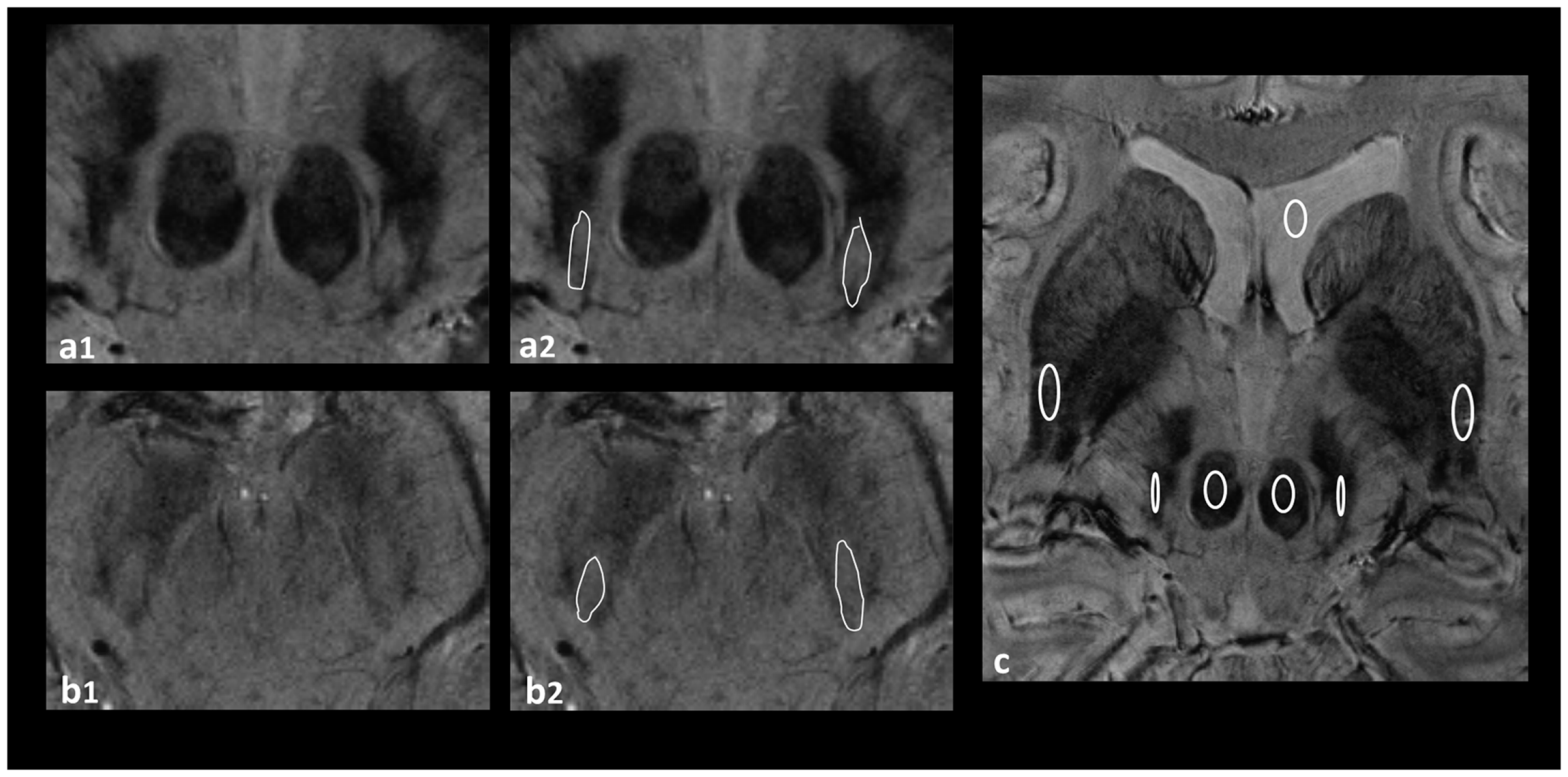
SWI 7T MRI of the SN. a_1_, b_1_: examples for a midbrain rated as bilaterally clearly visible nigrosome 1 (Group I); a_2_, b_2_: highlighted nigrosome 1 in the same subjects; c: ROI positioning in the SN, putamen, red nucleus and CSF of the ventricular system.

The signal intensities of the SN, putamen and red nucleus were measured within regions of interest (ROIs) placed on these three nuclei. Size of ROIs and positioning within the three nuclei were similar among all study subjects. The ROI placed in the SN was 15 mm^2^, in the putamen 44 mm^2^ and in the red nucleus 25 mm^2^. A reference measurement was carried out in the cerebrospinal fluid (CSF) of the ventricular system. To normalize the individual SIs according to differences in scaling during the image reconstruction process measurements of the SIs were divided by the individual reference measurement in the CSF. Fig 1 shows the appearance of a midbrain and the surrounding structures in 7T SWI. ROI-positioning is demonstrated in Fig 2c. Impairment by artefacts was rated visually on a scale from 1 to 5: 1 meant “image reading strongly impaired by artefacts”, 2 implied “image reading moderately impaired”, 3 meant “satisfactory image reading”, 4 stood for “good image reading” and 5 meant “no artefacts”.

### Statistical analysis

Data were tabulated and analyzed using GraphPad Prism (version 5.01). To assess age-dependent differences in visibility of the nigrosome 1 and in iron accumulation quartiles were used to divide the sample in four groups of approximately similar size (Group 1: 19–31 years, n = 11; Group 2: 32–43 years, n = 12; Group 3: 44–56 years, n = 12; Group 4: 57–75 years, n = 11). To verify the reproducibility of the findings regarding the visibility of nigrosome 1, inter-rater reliability between the two reviewers was calculated using Cohens kappa (κ) statistics. To assess the differences in SI-measurements for each nucleus among the four groups minimum, maximum, median, mean and standard deviation of the ratios for each nucleus and group were computed. Subsequently a Kruskal-Wallis Test was used, followed by a Dunn`s multiple comparison post test, when a significant difference was found. Finally, the ratios of the SI-measurements were plotted for each group and each nucleus in a scatter plot and those of subjects who were rated with at least unilateral “possibly visible nigrosome 1” were highlighted in order to determine their position within the group and in relation to the respective median and mean.

## Results

### Visibility of nigrosome 1

**Fig 3:**
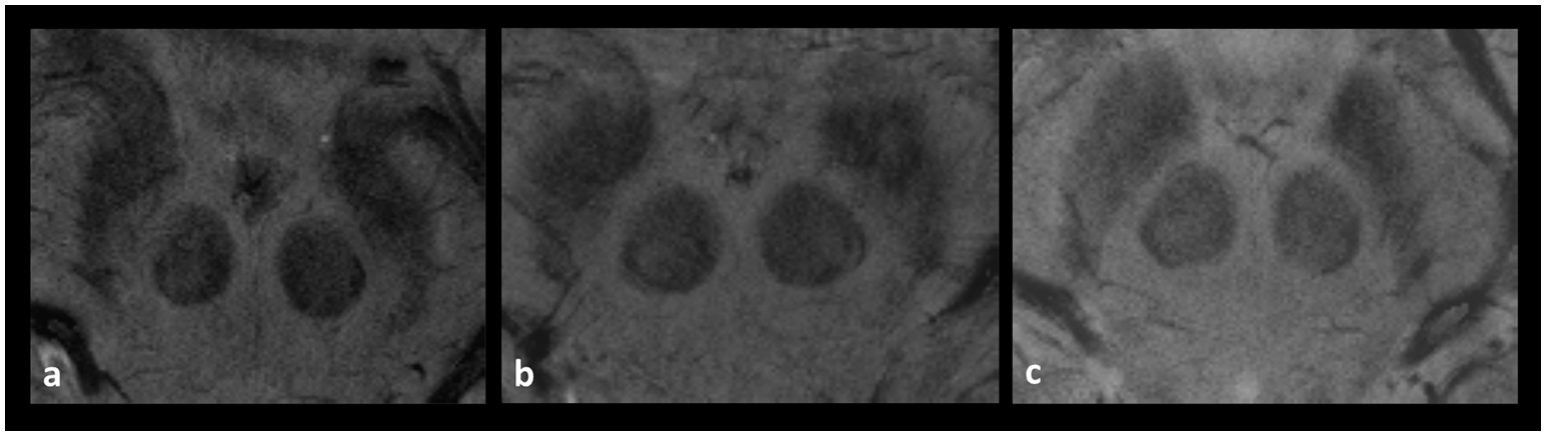
Midbrains rated as “limited visibility”. a: unilateral “limited visibility on the right side rated as 1; b: bilateral “limited visibility” on each side rated as 1, c: bilateral not visible nigrosome 1.

Agreement between both readers in evaluating the visibility of nigrosome 1, calculated by Cohens-Kappa, was good for both sides: (right hemi-mesencephalon к = 0.671; left hemi-mesencephalon к = 0.777). Overall, good to satisfactory image quality (degree of artefacts rated with at least 3) was seen for the evaluation of the dorsolateral nigral hyperintensity in 38 of the study participants (79%). Table 1 shows the visibility of the feature among the four age-related subdivisions of the sample based on the individual ratings (0: “not visible nigrosome 1”, 1: “not clearly delimitable” 2: “clearly present”). Furthermore, it demonstrates the distribution of the visibility classification I to III between the age-related subdivisions. The right column only includes those individual ratings per age-related subdivision indicating any limited visibility, i.e. ratings with at least a unilateral rating of 1 (“not clearly delimitable”) by at least one of the readers. Fig 2a_1_ and 2b_1_ show examples for midbrains rated as clearly visible nigrosome 1 and Fig 3a–3c demonstrate examples rated as “limited visibility”. It is found that in no case of the total sample both readers classified each hemi-mesencephalon as “not visible nigrosome 1” (0). Only in one subject each hemi-mesencephalon was rated as 0 by one observer, whereas the other observer rated both sides as 1 (“not clearly delimitable”). In the consensus rating the readers could agree on a unilateral absent and unilateral limited visibility. This subject with the worst rating was 45 years old and in the age-dependent group 3, close to the overall mean age of 44.5 years. Image quality has been rated as 3 („satisfactory“). The only other subject with a rating of 0 = “not visible nigrosome 1” has been rated by only one reader and only unilaterally. The other reader rated the hemimesencephalon of this subject as 1. In the consensus rating the readers could agree on a bilateral limited visibility. This subject was 49 years old and also in the age group 3, close to the overall mean age of 44.5 years, similar to the other subject. Image quality in this subject has been rated as 4 („good“). Overall, both readers classified 43 subjects (93%) as Group I (normal: nigrosome 1 was clearly present bilaterally or unilaterally and contralateral possibly present). That means that the specificity of this MR feature across all age groups reached approximately 94%. All subjects of group 1 and 4, i.e. the youngest and the oldest subjects of the sample were classified as Group I. Of the 2^nd^ group both readers classified the same one subject as Group II. One reader classified one subject of the 3^rd^ group as Group II and both readers classified the same one subject as Group III. However, in absolute values, numbers of subjects with at least unilateral limited visibility as tabulated in the right column of Table 1 differed markedly from the first to the 4^th^ group: Whereas of those subjects in group 1 only one was rated as such, one reader rated three and one reader even four subjects as “limited visibility” in the 4^th^ group. Overall, in 11 subjects (24%) at least one-sided limited visibility was observed by at least one of the two readers. In only three cases ratings of the two readers differed from each other so that consensus ratings had to be sought as shown in Table 1. We found a certain gender imbalance in favor of male subjects regarding those rated as “limited visibility”. Both of the lowest rated subjects as well as the majority of all subjects in the right column were male subjects (8/11 = 73%). Differences between the left and the right hemi-mesencephalon were less clear: “limited visibility” was assessed 7 times concerning the right and 9 times concerning the left hemi-mesencephalon.

**Table 1:** Visibility of nigrosome 1.

Group		I	II	III	Limited Visibility		S	A
					R	L		
								
**1**	O_1_	11	0	0	1	2	m	3
**19-31y**								
**n = 11**								
	O_2_	11	0	0	1	2	m	3
**2**	O_1_	11	1	0	1	1	f	5
**32-43y**					2	1	f	2
**n = 12**					2	1	m	3
	O_2_	11	1	0	1	1	f	5
					2	1	f	2
					2	1	m	3
**3**	O_1_	11	0	1	0	0	m	3*
**44-56y**					2	1	m	5
**n = 12**								
	O_2_	10	1	1	1	1	m	3*
					2	1	m	5
					0	1	m	4**
**4**	O_1_	11	0	0	2	1	m	3
**57-75y**					1	2	m	4
**n = 11**					2	1	m	4
	O_2_	11	0	0	2	1	m	3
					1	2	m	4
					1	1	f	4***
					2	1	m	4

* two different ratings: 0/0 resp. 1/1; consensus rating: 0/1

** two different ratings: 2/2 resp. 0/1; consensus rating: 1/1

*** two different ratings: 2/2 resp. 1/1; consensus rating: 1/1

O: observer; S: Sex; A: Artefact

I (normal): Nigrosome 1 was clearly present bilaterally or unilaterally and contralateral possibly present

II (non diagnostic): Nigrosome 1 was bilaterally possibly present

III (abnormal): Nigrosome 1 was absent unilaterally or bilaterally.

Limited visibility: number of subjects with at least unilateral rating as 1 by at least one observer

### Signal intensities

**Fig 4:**
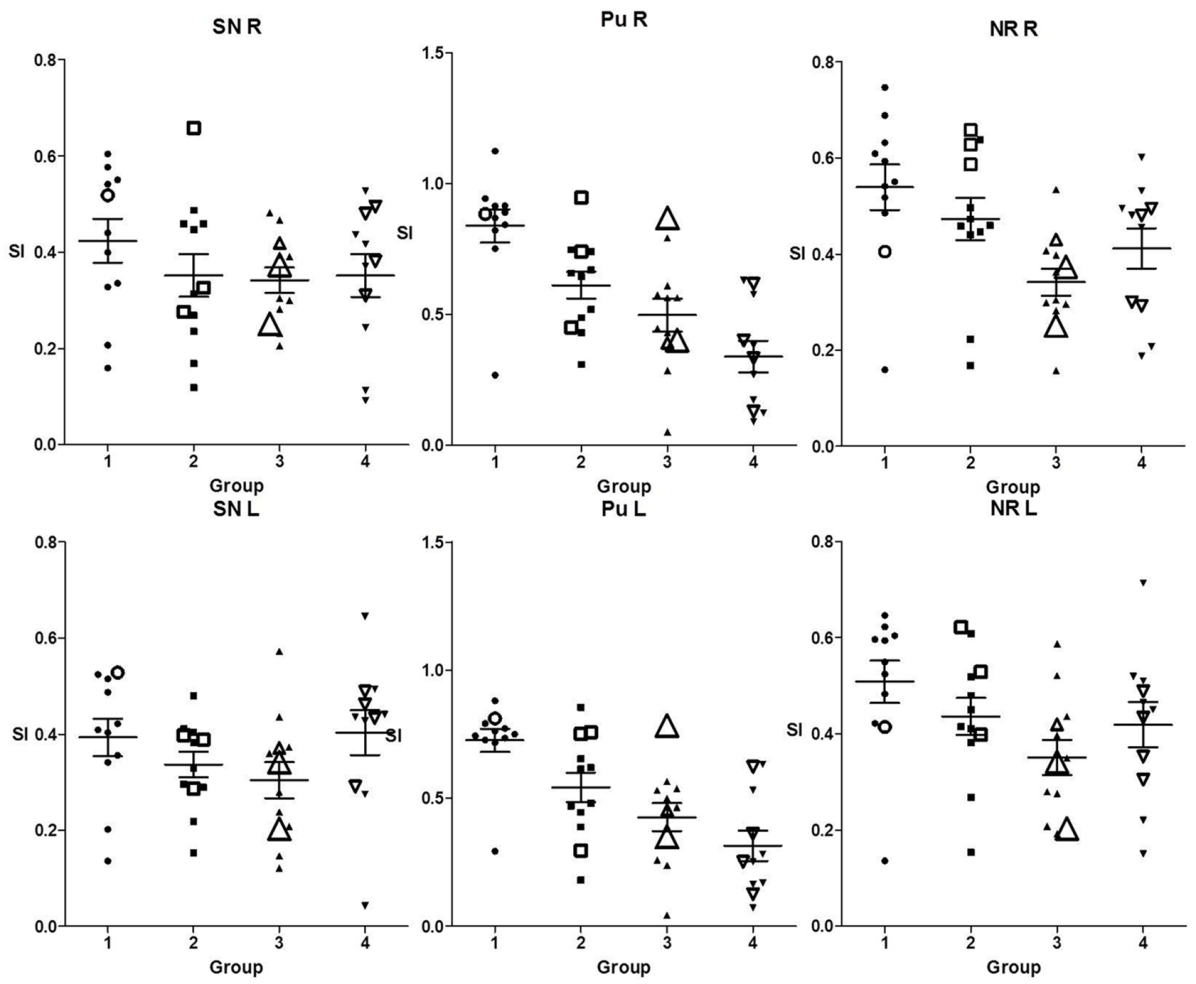
Column scatter plots of the SIs of all subjects within the respective nuclei and age groups. SIs of subjects rated as “limited visibility” are marked by size and shape: medium size = “limited visibility” with at least a unilaterally rated 1, biggest size = containing a rating as 0 “not visible nigrosome 1”.

Table 2 shows the descriptive statistics of the SI-measurements for each nucleus among the four groups. In the first row results of the Kruskal-Wallis Test are demonstrated and in case of significance results of Dunn`s multiple comparison post test are captured below the table. In Fig 4 results of Tables 1 and 2 are combined. The ratios of the signal intensities are plotted for each age-related group in column scatter plots and those of subjects with limited visibility are marked by size and shape. Table 2 as well as Fig 4 show that the mean SIs as markers for iron-accumulation decreased from group 1 to group 3 in all three nuclei and showed a mild increase from group 3 to 4 in the SN and red nucleus which could not been detected in the putamen. However, the Kruskal-Wallis Test pointed out that the differences between the age groups were not significant in the SN, while the largest differences concerned the putamen. More precisely the post test revealed the overall largest group differences between group 1 versus group 4 concerning the putamen and lower but also significant differences between group 1 and 3 concerning the red nucleus. The SIs of the two subjects with the worst ratings concerning the visibility of nigrosome 1 as well as SIs of those rated with “limited visibility” were scattered among the age groups and partly close to the median SI of each nucleus in the respective age group and partly near the upper or lower limits of the plot.

**Table 2:** Normalized SIs per age group and nucleus.

Group		SN R	SN L	Pu R	Pu L	NR R	NR L
p^a^		-	-	***	***	**	*
							
**1**	Minimum	0.1596	0.1362	0.2681	0.2936	0.1596	0.1362
**19-31y**	Maximum	0.6046	0.5283	1.125	0.8807	0.7471	0.6462
**n = 11**	Median	0.4401	0.4091	0.8840	0.7501	0.5505	0.5494
	Mean	0.4238	0.3931	0.8387	0.7260	0.5392	0.5084
	SD	0.1516	0.1292	0.2106	0.1507	0.1573	0.1462
**2**	Minimum	0.1188	0.1535	0.3094	0.1807	0.1683	0.1535
**32-43y**	Maximum	0.6582	0.4803	0.9460	0.8542	0.6582	0.6212
**n = 12**	Median	0.3203	0.3565	0.6513	0.5470	0.4663	0.4325
	Mean	0.3517	0.3366	0.6120	0.5426	0.4730	0.4361
	SD	0.1539	0.09206	0.1768	0.2010	0.1526	0.1332
**3**	Minimum	0.2058	0.1213	0.05070	0.04374	0.1581	0.1918
**44-56y**	Maximum	0.4825	0.5720	0.8684	0.7842	0.5343	0.5866
**n = 12**	Median	0.3390	0.3109	0.5050	0.4586	0.3335	0.3458
	Mean	0.3419	0.3042	0.4985	0.4263	0.3415	0.3508
	SD	0.09214	0.1299	0.2183	0.1895	0.09754	0.1268
**4**	Minimum	0.09192	0.04271	0.07221	0.07221	0.1878	0.1510
**57-75y**	Maximum	0.5271	0.6456	0.6330	0.6330	0.6018	0.7135
**n = 11**	Median	0.3811	0.4356	0.2519	0.2519	0.4801	0.4504
	Mean	0.3515	0.4034	0.3150	0.3150	0.4118	0.4190
	SD	0.1478	0.1552	0.1980	0.1980	0.1394	0.1555

P^a^: P value of Kruskal-Wallis-Test for the comparison of the same nucleus in all groups:

**SN R** = 0.3886; **SN L** = 0.0929; **Pu R** = 0.0001; **Pu L** = 0.0003; **NR R** = 0.0062;

**NR L** = 0.0419

Dunn`s post Test (P<0.05):

**Pu R** Group **1** vs **3**:**, **Pu R** Group **1** vs.**4**:***

**Pu L** Group **1** vs **3**:*, **Pu L** Group **1** vs.**4**:***

**NR R** Group **1** vs. **3**:**

**NR L** Group **1** vs. **3**:*

## Discussion

Since 2013 ten studies [7, 9, 12–19] and one meta-analysis [26] have been published dealing with a hyperintense area within the dorsolateral border of the otherwise hypointense SNc on iron-sensitive MR imaging sequences as a diagnostic marker. This structure of the midbrain is referred to as nigrosome 1 [9, 13, 15, 19], (dorsolateral) nigral hyperintensity [14, 18], “substantia nigra hyperintensity” [17], “intermediate hyperintense SN component” or “hyperintense oval area between two hypointense layers” in the same study [12] or swallow tail appearance [7] /sign [16] alternatively and in the present study as nigrosome 1 visibility. Previously mentioned studies so far have largely focused on the absent visibility of nigrosome 1in patients with PD or additionally in patients with MSA and PSP [14]. Assessment of the appearance in healthy subjects has been predominantly limited to the examination of small healthy control groups, lower field strength or T2* weighted imaging instead of SWI.

Only three 7T studies dealing with this subject have been published until now [9, 12, 17]. In two of them [9, 17] T2*-weighted sequences were used as an iron-sensitive MR technique. According to the results of previous studies, T2* weighted imaging is up to three-times less sensitive for iron deposits than SWI Imaging [27–29]. In the only other previous study performed on a 7T MR system and using susceptibility weighted imaging [12] only 13 healthy subjects (mean age, 54.7 years; age range, 41–66) were examined.

Seven other studies were performed on 3T MR systems [7, 13–16, 18, 19]. They used either SWI [7, 14–16, 18] or a MEDIC-sequence [13, 19] -a heavily T2* weighted spoiled gradient echo sequence with multiple echoes.

The current study systematically investigates the visibility of nigrosome 1in 46 subjects without neurodegenerative disorders such as PD using 7T UHF MR imaging with a SWI sequence. Furthermore, in this work for the first time, the visibility of this substructure of the SN has been examined and compared between four age groups of healthy subjects within a sample covering over five decades in order to evaluate the impact of an assumed increasing iron-accumulation with age on the visibility.

The results of our study indicate that nigrosome 1 can be detected relative robustly in subjects without PD using 7T UHF MR imaging and a SWI sequence which is congruent to the results of the control groups examined in the 10 aforementioned studies [7, 9, 12–19]. Additionally, our findings suggest that it is a fairly dependable imaging marker among all age groups. In terms of the classification of Schwarz et al. [7] the overall specificity of the nigrosome 1 visibility reached approximately 94% as 43 subjects from a total of 46 could be classified as Group I (Group I = normal: nigrosome 1 was clearly visible bilaterally or unilaterally and contralateral possibly visible). According to this high specificity our results correlate very well with a previously published meta-analysis [26] dealing with eight of the mentioned studies [7, 9, 12–14, 17–19] and one using FLAIR-sequences [30]. However, there are still debatable limits. In 24% at least one-sided “limited visibility” was detected, which, according to the former studies must be interpreted as suspected PD. This exceeds the assumed prevalence of PD of 257/100000 = 0.26% [31] by far. Therefore the impact of the nigrosome 1 visibility has to be further examined in larger healthy cohorts. However, in comparison to the mentioned previous studies we performed a more detailed gradation, ranging from “one sided possible visibility” to “both sided absent visibility”. In this context, we would like to point to the possibility that the two cases with at least one hemi-mesencephalon rated as “not visible nigrosome 1” (0) and maybe even all cases with limited visibility constitute preclinical stages of a future PD. Idiopathic rapid eye movement (REM) sleep behavior disorder (iRBD) is a parasomnia that in the majority of the cases exhibits a conversion to neurodegenerative diseases, most frequently PD [32]. This means that a proportion of patients with iRBD represents prodromal stages of PD. De Marzi et al. could show that two thirds of iRBD patients showed loss of dorsal nigral hyperintensity (DNH) [33]. This observation suggests that limited visibility of nigrosome 1 might not only be a diagnostic biomarker for symptomatic PD but rather also for prodromal PD. Thus, it could be interesting to assess these cases of limited visibility for subtle, subclinical signs of PD and follow them up clinically.

Our hypothesis of a decreasing visibility with age could not be verified in terms of the classification of Schwarz et al. [7] because 93% of the volunteers were classified as “normal” (Group I) and in particular all subjects in group 4, i.e. the oldest subjects of our sample were all classified as “normal”. Furthermore, the age of the two subjects unilaterally rated as “absent visibility” was very close to the mean (44.5 y) of the sample rather than near the maximum age as expected. However, differences which could be possibly interpreted as age-dependent were found in detection reliability of nigrosome 1. From the group with the youngest subjects to the one with the oldest, the number of subjects rated as “limited visibility” increased from one per observer (1/11) to four per observer (4/11). However, we should point out that the number of subjects in each group was small (11 subjects in group 1 and 4 and 12 in group 2 und 3) so that these fine gradations in the classification of the visibility might lead to weak generalizing explanatory power.

The non-heme iron concentration in the brain is greater than in any other organ system except the liver [34, 35] but the distribution varies across brain regions. In circuits associated mainly with the domain of movement the largest amounts of iron can be found [35]. Postmortem studies could measure the highest concentrations in the basal ganglia [36, 37]. Furthermore, non-heme iron is not only unevenly distributed across brain regions but also the iron concentrations show substantial age differences. The magnitude of these age differences varies among different regions [38, 39] and is most pronounced in the basal ganglia [34, 36, 39]. Thus, we hypothesized that increasing brain iron accumulation in the aging brain might lead to a decreasing visibility of nigrosome 1 also in subjects without PD. Indeed, our assessment of the SIs measured among different age groups and nuclei reflects the results of previous studies. The mean SIs as markers for iron-accumulation decreased with age in all three nuclei but differences between the age groups were not significant in the SN. These findings are in agreement with studies that could not find any significant changes in SN iron content as a function of age [34, 40–43] and with those showing a rapid increase in the first two and inconclusive results for later decades [34] or describing that the iron content reached a plateau after the age of 15 [41]. In our study the largest differences of SIs between age groups could be detected in the putamen. This conforms to previous reports using iron-sensitive MRI techniques and showing that iron concentration in the putamen was age dependent [40, 43–50] and did not reach its maximum until the sixth decade [34, 40]. Finally, our results show lower but significant differences of SIs between age groups in the red nucleus with a decrease from group 1 to 3 and a mild increase from group 3 to 4. Similar nonlinear age trends in this nucleus have been reported in several studies [44, 47, 51], one of them noting an” increase of susceptibility until mid-life, followed by a brief plateau before a slight decay from the 6th decade on” [44]. In summary our results concerning SIs representing correlates of iron concentrations are in line with previous reports and confirm observations about increasing brain iron accumulation in some structures of the aging brain. However, our hypothesis that these changes might lead to a decreasing visibility of nigrosome 1 also in subjects without PD has to be rejected for two main reasons: firstly because within the respective age groups and nuclei SIs of subjects rated as “limited visibility” were scattered and not primarily found near the minimum and secondly because precisely the SN showed no significant age dependent differences in iron concentrations. As demonstrated in Fig 4 within the four age-dependent plots of signal intensities measured in the SN even more subjects rated as “limited visibility” could be found above the respective mean and not as could have been expected below it.

The findings of the present study should be interpreted in the context of its limitations. First, the sample size was small. Although the number of healthy subjects examined in this study clearly exceeded those examined in the only other 7T study using SWI-sequences [12] number of subjects in a single age group remained small. Thus, it is debatable, if an impact of aging on the visibility can be completely ruled out or, if the increase of the number of subjects rated as “limited visibility” from group 1 to 4, as well as the observation that the two subjects with the worst ratings were found in group 3, show a real trend toward decreasing visibility of nigrosome 1 with age. Second, because of the retrospective characteristic of the study we could only evaluate images with a slice-thickness of 1mm instead of submillimeters. Furthermore, we would like to state that the determinants of iron deposition during normal brain aging are relatively unknown [52] and that we cannot ensure, that the observed trends in signal intensity changes of our relative small sample are incurred solely as a result of aging. Finally, we cannot prove that the signal of the dark structures of the SN is exclusively induced by iron. Other substances such as myelin and calcification may also cause a change in regional susceptibility value. Nevertheless, there is an emerging consensus that the paramagnetic susceptibility of gray matter is mainly related to iron [53] and our key result would not have been influenced by other factors contributing to increasing SIs for two reasons: first, SIs in the SN did not differ significantly among the four age groups and second, we could not find a correlation between lower SIs and decreasing visibility of nigrosome 1.

## Conclusions

The results of the current study support the potential of the assessment of the nigrosome 1 visibility as a possible future differential diagnostic tool to distinguish between healthy subjects and patients with PD. Furthermore, the results suggest, that normal aging with a related increase in iron concentrations in the basal ganglia does not affect the visibility of nigrosome 1. However, further qualitative and quantitative analyses are necessary to determine the characteristics of its visibility. Future larger studies should test the normal spectrum of this MR feature as it occurs in subjects without PD and further large-scale prospective studies are needed to better define the extent of a “limited visibility” to which an individual can be considered healthy. Finally, more studies are needed to define optimal imaging solutions in terms of field strength, slice-thickness and orientation. Without the development of reproducible and standardized MR-protocols as well as systematic evaluation systems of the nigrosome 1 visibility a dependable diagnostic marker is currently an unmet need.

## Supporting information

S1 File Raw data set with individual ratings, signal intensities and characteristics of the subjects. (XLSX)

## Abbreviations

APD: atypical parkinsonian disorders
CSF: cerebrospinal fluid
DNH: dorsal nigral hyperintensity
iRBD: idiopathic rapid eye movement sleep behavior disorder
MSA: multisystem atrophy
N1: nigrosome 1
PD: Parkinson`s disease
REM: Rapid eye movement
RF: radiofrequency
ROI: region of interest
SD: standard deviation
SI: signal intensity
SN: substantia nigra
SNc: pars compacta of the substantia nigra
SNr: pars reticulata of the substantia nigra
STS: swallow-tail-sign
SWI: susceptibility weighted images
TE: echo time
TR: repetition time
UHF: ultrahigh field
